# Personalized Precision Medicine for Health Care Professionals: Development of a Competency Framework

**DOI:** 10.2196/43656

**Published:** 2023-02-07

**Authors:** Fernando Martin-Sanchez, Martín Lázaro, Carlos López-Otín, Antoni L Andreu, Juan Cruz Cigudosa, Milagros Garcia-Barbero

**Affiliations:** 1 Department of Biomedical Informatics and Digital Health National Institute of Health Carlos III Madrid Spain; 2 Department of Medical Oncology, University Hospital Complex of Vigo Vigo Spain; 3 Department of Biochemistry, University of Oviedo Oviedo Spain; 4 European Infrastructure for Translational Medicine Amsterdam Netherlands; 5 Department of University, Innovation and Digital Transformation, the Government of Navarra Navarra Spain; 6 Faculty of Medicine, Miguel Hernández University Alicante Spain

**Keywords:** personalized precision medicine, professional competence, domains, determinants of health, digitalization, communication, bioethics, digital health

## Abstract

**Background:**

Personalized precision medicine represents a paradigm shift and a new reality for the health care system in Spain, with training being fundamental for its full implementation and application in clinical practice. In this sense, health care professionals face educational challenges related to the acquisition of competencies to perform their professional practice optimally and efficiently in this new environment. The definition of competencies for health care professionals provides a clear guide on the level of knowledge, skills, and attitudes required to adequately carry out their professional practice. In this context, this acquisition of competencies by health care professionals can be defined as a dynamic and longitudinal process by which they use knowledge, skills, attitudes, and good judgment associated with their profession to develop it effectively in all situations corresponding to their field of practice.

**Objective:**

This report aims to define a proposal of essential knowledge domains and common competencies for all health care professionals, which are necessary to optimally develop their professional practice within the field of personalized precision medicine as a fundamental part of the medicine of the future.

**Methods:**

Based on a benchmark analysis and the input and expertise provided by a multidisciplinary group of experts through interviews and workshops, a new competency framework that would guarantee the optimal performance of health care professionals was defined. As a basis for the development of this report, the most relevant national and international competency frameworks and training programs were analyzed to identify aspects that are having an impact on the application of personalized precision medicine and will be considered when developing professional competencies in the future.

**Results:**

This report defines a framework made up of 58 competencies structured into 5 essential domains: determinants of health, biomedical informatics, practical applications, participatory health, and bioethics, along with a cross-cutting domain that impacts the overall performance of the competencies linked to each of the above domains. Likewise, 6 professional profiles to which this proposal of a competency framework is addressed were identified according to the area where they carry out their professional activity: health care, laboratory, digital health, community health, research, and management and planning. In addition, a classification is proposed by progressive levels of training that would be advisable to acquire for each competency according to the professional profile.

**Conclusions:**

This competency framework characterizes the knowledge, skills, and attitudes required by health care professionals for the practice of personalized precision medicine. Additionally, a classification by progressive levels of training is proposed for the 6 professional profiles identified according to their professional roles.

## Introduction

In 2019, the National Health Service published the Topol report identifying key areas for addressing the health care challenges of the 21st century. This report concludes that “educating the current and future health care professionals is key to enabling the implementation of the revolutionary changes in clinical practice and medical care that technological advancement will bring for the benefit of patients, caregivers, and citizens” [1].

The growing and continuous incorporation of new knowledge and technologies poses major challenges to health care and health care professionals who must continuously update their practice. Due to scientific advancements, training is a fundamental pillar for implementing new competencies; therefore, creating an environment of continuous learning has become essential to respond to the demands of the population and place the patient at the center of the system.

Personalized precision medicine is an emerging field of medicine that addresses the prevention, diagnosis, and treatment of diseases by considering individuals’ genetic and genomic data, clinical data, and environment [2,3]. It represents a paradigm shift in health care and a new reality for the health care system that favors the use of more effective and safer preventive, diagnostic, and therapeutic health interventions and contributes to the sustainability of the health care system. However, the full incorporation of personalized precision medicine and its application in clinical practice raises important training challenges for health care professionals who will need to acquire competencies aimed at performing their professional practice in an optimal, efficient, and quality manner in the Spanish health care system [4,5].

In Article 42 of the Spanish Law on Cohesion and Quality of the National Health System, competency is defined as “the aptitude of the health care professional to integrate and apply the knowledge, skills, and attitudes associated with the good practices of his or her profession to resolve the situations that arise” [6]. In this context, the acquisition of competencies by health care professionals can be defined as a dynamic and longitudinal process.

Accordingly, this project aimed to define a proposal of common domains and competencies for today’s health care professionals, as well as those who will emerge in the future [7]. This competency framework will also serve as a support instrument for the implementation of programs and initiatives aimed at the training and certification of health care professionals working in personalized precision medicine. It will also facilitate the development and accreditation of training content and educational programs, among other applications. 

## Methods

### Overview

The methodology of this project took a broadly participatory and multidisciplinary approach in line with the nature of personalized precision medicine, wherein different areas of knowledge and professionals participated in its development and complete definition.

Two groups of experts were set up: a working group and an expert group. The working group was composed of 6 experts who analyzed the articles and reports of interest, helped identify competency frameworks and training programs, expressed opinions and issued recommendations on different aspects of the framework, and reviewed and validated the documents. The professional profiles represented in the working group are detailed in Textbox 1.

The expert group included 11 experts from different fields of knowledge including determinants of health, bioinformatics, bioethics, and other disciplines involved in personalized precision medicine. These experts, through individual interviews, helped us identify the areas of knowledge and competencies to be developed or acquired by health care professionals working in the field of personalized precision medicine. The professional profiles of the individuals included in the expert group are detailed in Textbox 2.

**Field of expertise and professional profiles of the working group.**

1. Academic: professor of biochemistry and molecular biology
2. Academic: professor of health systems
3. Public health research institute: research professor of biomedical informatics
4. Hospital clinician: medical oncology
5. Government: digital transformation and innovation
6. Research: translational medicine

**Professional profiles and field of expertise of the expert group.**

1. Public health research institute: research professor biomedical informatics
2. Hospital clinician: psychiatry and mental health
3. Academic: bioethics
4. Public health research institute: oncology/genetics
5. Public health research institute: environmental health
6. Government: humanization and social health care
7. Academic: pharmacogenetics and pharmacogenomics
8. Public health research institute: medical oncology
9. Hospital clinician: rheumatology
10. Academic: computer science and artificial intelligence
11. Academic: medical education

### Benchmark Analysis: Competency Frameworks and Training Programs

The objectives of the benchmark analysis were to identify and analyze documents that could be used to conceptualize the structure of the framework and identify possible competencies. To achieve these goals, we had the support and expertise of Ascendo Sanidad&Farma [8], a strategic and operations consulting firm that specializes in the health care sector. The consulting team gathered all the information and carried out a detailed analysis of the documents identified by the working group. To conceptualize the structure of the framework and the areas of knowledge, a total of 61 documents were identified, of which 22 (36%) documents covering competency frameworks and training programs of reference were identified and analyzed [4,5,9-28]. Among them, 4 (18%) covered transversal competencies for health professionals, 8 (36%) referred to competency frameworks in digital health, and 5 (23%) referred to competency frameworks in genetics and genomics. In addition, 4 (18%) training programs in the field of personalized precision medicine were included in the analysis. The remaining 39 (64%) documents consisted of relevant articles and reports that were identified by the working group [1,29-66]. The aim was to identify and determine areas of knowledge that could constitute the different domains of the competency framework and highlight key aspects that are currently impacting the application of personalized precision medicine in clinical practice.

### Workshop 1: Consensus on Key Elements and Training Needs

The information and conclusions drawn from this analysis, together with the contributions of the members of the working group, allowed us to identify a series of essential domains for all health care professionals working in the field of personalized precision medicine. This identification enabled us to reach a consensus on the structure of the competency framework, considering a total of 6 domains, and to carry out a preliminary identification of the main lines to be addressed within each domain in the form of competencies. We also determined key elements and training needs for the development of skills in the areas of interest for personalized precision medicine.

### Interviews

Individual interviews were carried out with the expert group to identify competencies for each of the 6 already defined domains in the first phase, as well as to relate those competencies with different professional profiles to facilitate their work in personalized precision medicine. Based on the information obtained in the analysis of documents and the vision provided by the experts in the interviews, an initial proposal of competencies for health care professionals in this field was made.

### Workshop 2: Consensus on Areas of Knowledge/Essential Domains and Common Minimum Skills

The second workshop aimed at reaching a consensus on the competencies identified during the interviews and through the literature review. Additionally, in this workshop, 6 generic health care professional profiles were defined based on the different subdisciplines and tasks in which they develop their professional activities.

For each professional profile identified, a simple classification of progressive levels of development according to the degree of depth that a professional should acquire for each competency was established. In this sense, based on the Bloom taxonomy, a method commonly used for establishing curriculum learning objectives [67], we determined 3 levels of knowledge (basic, intermediate, and advanced) for each professional profile.

### Ethical Considerations

This competency framework was developed based on a benchmark analysis of other competency frameworks and training programs related to the field of personalized precision medicine that are publicly available. Additionally, all contributions made by groups of experts that participated were made with their permission and authorization. Furthermore, no personal data of any kind were collected to conduct this work. Therefore, no independent ethical approval was required for the development of this study.

## Results

### Benchmark Analysis Results: Competency Frameworks and Training Programs

The selection and analysis frameworks of competencies and training programs of reference developed by scientific societies and other organizations allowed us to identify the training needs generated by the emergence of new areas of knowledge, such as digital health or genomics, and thus establish the foundation for the definition of the competency framework.

After analyzing the documents and collecting the opinion from the expert group, 12 general conclusions were reached (described in Textbox 3) for the development of a competence framework in personalized precision medicine in Spain.

**Conclusions of the analyzed documents.**

1. There are several examples of general competency frameworks for health care professionals that are intended to guide the design of training programs. In general, these frameworks include both professional competencies (eg, knowledge of scientific and clinical fundamentals) and cross-cutting competencies (eg, communication, leadership, management, and collaboration skills) focused on professional values and skills.
2. In general, competency frameworks are structured in competency domains, and some also classify competencies according to their level or degree of specialization and the professional profile to whom they are addressed.
3. Regarding digital health and health informatics, numerous examples of competency frameworks for health care professionals were identified. Generally, competency frameworks include health and biomedical science competency domains (eg, health systems), technological competencies in the use of informatics tools, competencies in the use and management of data (including aspects related to data security and protection), and cross-cutting competencies (eg, ethics, management, leadership, communication, and collaboration).
4. In the field of genomics, several competency frameworks aimed at different profiles of health professionals were identified. The competency frameworks analyzed go beyond basic knowledge in this area, with a focus on the analysis and interpretation of results, aspects related to information management and communication to patients, and other ethical, legal, and social aspects.
5. Most of the identified competency frameworks, despite being focused on a specific field of knowledge (eg, digital health or genomics), in most cases incorporate more cross-cutting competencies, such as communication, strategy, research, bioethics, leadership, change management, and governance.
6. At the European level, there are examples of training programs in personalized precision medicine, such as the European Infrastructure for Translational Medicine (EATRIS) summer school in personalized medicine, the Personalized Medicine Inquiry-Based Education (PROMISE), the European Region Action Scheme for the Mobility of University Students Plus Programme (ERASMUS+), and the Bridge Translational Excellence Programme of the University of Copenhagen. These programs combine both training elements in clinical and basic research, as well as cross-cutting knowledge and skills (eg, communication and patient engagement, ethics, management, and leadership in translational medicine).
7. At the national level, the Integrated Strategy for Personalized Medicine in Navarra, Spain, highlights the need to have specific competencies in personalized precision medicine for professionals in different fields. To achieve this objective, one of the axes of this strategy focuses on training in areas identified as relevant in the field of personalized precision medicine: genomics and multiomics, information and communications technology (ICTs) and digital health, bioinformatics, data science, ethical-legal regulations and data protection, evaluation of scientific evidence, and research methodology.
8. Personalized precision medicine is a key element of the medicine of the future and, in combination with the development of digital tools and artificial intelligence techniques, will make it possible to combine clinical, genomic, and environmental information (social and environmental determinants of health) to improve the planning of therapeutic, preventive, and diagnostic strategies.
9. Genomics, digital medicine, artificial intelligence, and robotics are key areas to address health care challenges of the future. Therefore, educating current and future health care professionals in these areas is critical to enable the implementation of the revolutionary changes expected for clinical practice and health care in the future.
10. Addressing the future challenges of medicine requires a shift from the traditional disease-free approach to a health-oriented medicine that holistically addresses all aspects of an individual's health.
11. Based on the current definitions of health and personalized precision medicine, as well as its translation to clinical practice, several areas and knowledge need to be considered to achieve an optimal future for medicine that responds to the needs of each individual.
12. Once the analysis was carried out, the importance of considering areas of knowledge, such as genomics and other omic sciences, digital medicine, tools for management, interpretation, and support for decision-making based on data (eg, artificial intelligence) as well as general aspects, such as multidisciplinary work, leadership, and ethical and safety conditions, became clear.

### Structure of the Competency Framework and Professional Profiles

**Figure 1:**
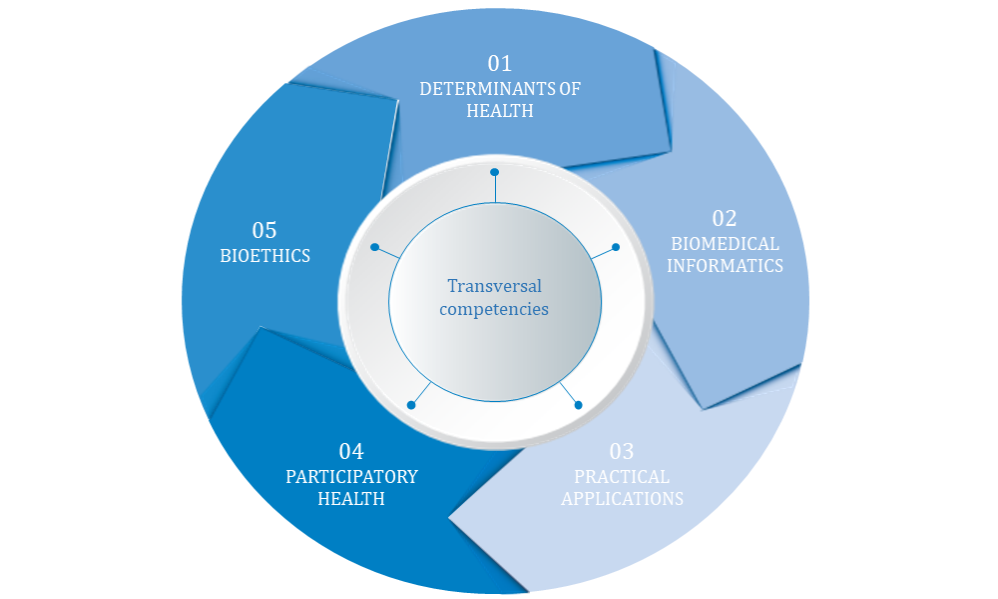
Structure of the competency framework for healthcare professionals in personalized precision medicine.

Following the analysis of the most relevant competency frameworks and training programs, interviews with experts, and workshops held with the working group, the competency framework’s structure was defined. This competency framework will respond to the needs and challenges posed by the complete incorporation of personalized precision medicine (Figure 1).

The framework is structured into 5 essential domains: determinants of health, biomedical informatics, practical applications, participatory health, and bioethics, with an additional sixth cross-cutting domain that impacts the overall performance of the competencies linked to each of the previous domains (Textbox 4).

Within these domains, it was essential to define the health care professional profiles to which this proposal of competencies is addressed. In this sense, although new profiles and professionals will emerge with scientific advancements, 6 generic professional profiles were identified based on their professional activity within personalized precision medicine (Textbox 5).

**Classifications and descriptions of the 6 domains.**

1. Determinants of health: It includes competencies that enable health care professionals to take a holistic approach that considers biological, environmental, and other determinants of health within the framework of personalized precision medicine.
2. Biomedical informatics: It includes competencies that enable health care professionals to develop their activity by considering technical and practical aspects of the digital transformation of the health care system, digitization, and other related tools for the full incorporation of personalized precision medicine.
3. Practical applications: It includes competencies that enable health professionals to develop strategies based on personalized precision medicine, both at individual and community levels, for the prevention, diagnosis, treatment, and follow-up of the disease.
4. Participatory health: It includes competencies that enable health care professionals to promote patient participation by considering their needs and preferences and ensuring respectful, empathetic, and individualized communication.
5. Bioethics: It includes competencies that enable health care professionals to apply the principles of bioethics in the practice and development of personalized precision medicine.
6. Transversal competencies: It includes competencies that have an impact on the general performance of the competencies linked to the other 5 domains, helping health care professionals to perform their professional work optimally in the field of personalized precision medicine and the health system.

**Professional profile classification based on professional activity.**

1. Clinical: health care professionals who carry out their work in the field of health care in contact with patients (primary care and secondary care)
2. Laboratory: health care professionals who work in the laboratory or other units of a health care center without direct contact with patients
3. Digital health: all the new professional profiles arising from the digital transformation of the health care system
4. Community health: professionals working in the field of public health
5. Research: professionals who work in research in the field of personalized precision medicine
6. Management and planning: professionals working in positions with responsibilities for health care management and planning

### Proposal of Competencies in Personalized Precision Medicine for Health Care Professionals

Each of the 6 defined domains includes a series of competencies that health care professionals should acquire to guarantee the optimal development of their practice in the field of personalized precision medicine. In total, the competency framework includes 58 competencies (Tables 1-6).

**Table 1:** Proposal of competencies for domain 1: determinants of health.

Subdomains		Areas of competence
**Biological determinants**		
	D1.1	Principles of the molecular and pathophysiological basis of diseases from the perspective of the omic sciences
	D1.2	Principles of the different omics sciences, their current field of application (clinical/research field), and their advantages and limitations
	D1.3	Sources and types of data that can be obtained with the different omics technologies available and what information can be provided by each
	D1.4	Information derived from the study of omics data and its clinical and/or epidemiological implications
**Environmental determinants**		
	D1.5	Principles of environmental toxicology and environmental risk factors with an impact on health
	D1.6	Environmental behavior of chemical contaminants and environmental radiation
	D1.7	Most common routes and pathways of exposure and the tools to apply this information to an individual (exposome)
	D1.8	Bioaccumulation and biomagnification of pollutants along the trophic chain and their metabolism to understand how they reach individuals and how to interpret possible related findings
	D1.9	Prediction and evaluation of risks from environmental determinants to include them in decision-making
**Other health determinants**		
	D1.10	Use of the psychosocial model in the evaluation of the individual, including psychological, socioeconomic, and cultural factors, as well as habits and lifestyles and not only biological and environmental determinants

**Table 2:** Proposal of competencies for domain 2: biomedical informatics.

Subdomains		Areas of competence
**Data collection**		
	D2.1	Differences between data, information, and knowledge and their relationship
	D2.2	Most relevant sources and types of data in the field of personalized precision medicine, as well as the information that each of them can provide
	D2.3	Primary and secondary use of health data and main databases, along with their applications in the specific areas of activity
	D2.4	Strategies to improve data quality
	D2.5	Data life cycle and the importance of complying with FAIR^a^ principles to enable its use
	D2.6	Sharing of data, information, and knowledge generated within the framework of personalized precision medicine, as well as the main national and international initiatives in health data management
**Data management**		
	D2.7	Mechanisms to guarantee confidentiality, protection, and security and/or maintain anonymity in the storage of health data and information, ensuring the right to privacy and making appropriate use of the information
	D2.8	Most common data storage resources (centralized/federated databases) and the possibilities offered by each
	D2.9	Main ontologies and normalization standards in the field of health that would facilitate interoperability and data exchange
	D2.10	Incorporation of information in the electronic health record in an appropriate manner, ensuring its quality to guarantee that it is subsequently used
	D2.11	Legislative framework on the use and management of sensitive data and digital rights: the European regulation GDPR^b^ and the national regulation OLPDPGDR^c^ [68,69]
**Data analysis and interpretation of information**		
	D2.12	Methodologies available to perform data analysis: how the analysis is performed, the difficulties and limitations it presents, the quality of the data, etc
	D2.13	Software available for use in current clinical practice
	D2.14	Programming languages in health data analysis
	D2.15	Main technological trends that would be more important in the immediate future (eg, artificial intelligence, big data, Internet of Things, etc)

^a^FAIR: Findability, Accessibility, Interoperability, and Reusability.

^b^GDPR^:^ General Data Protection Regulation.

^c^OLPDPGDR: Organic Law 3/2018 on Personal Data Protection and Guarantee of Digital Rights.

**Table 3:** Proposal of competencies for domain 3: practical applications.

Subdomains		Areas of competence
**Individual interventions**		
	D3.1	Updating of knowledge and advances generated in the field of personalized precision medicine, especially those specific to this field of work
	D3.2	Available technologies linked to the collection of omic data to select the most appropriate one, depending on the information that needs to be obtained, the pathology, and the phase of the care process the patient is in
	D3.3	Databases for the correct clinical interpretation of the results derived from the omic tests performed
	D3.4	Process to reach a conclusion or recommendation from the interpretation of health data analysis as a support tool for clinical decision-making
	D3.5	Diagnostic, prognostic, and treatment biomarkers that allow stratification of patients, especially those biomarkers specific to its fields of work
	D3.6	Predictive biomarkers for the design of the individualized therapeutic plan considering the therapies associated with the expression of each of the biomarkers and the clinical situation of the patient
	D3.7	Determinants of the pharmacogenetic phenotype, pharmacological interactions, and drug response to optimize the design of the individualized therapeutic plan
	D3.8	Clinical decision support systems based on artificial intelligence and designed from the evidence generated from the analysis of large amounts of data
	D3.9	Personalized habit and lifestyle measures and recommendations based on the individual's environmental exposures and risk assessment
	D3.10	Existing tools to apply a family approach in those clinical situations or patients who require it
	D3.11	Genetic counseling based on the results of genetic analysis and the individual's situation, recognizing the implications derived from these analyses in terms of limitations, family repercussions, unexpected findings, and possible interventions in prevention and taking into consideration the ethical and legal derivations of this practice
**Precision community interventions**		
	D3.12	Precision health based on the design of actions to promote and maintain population health based on data, information, and analysis derived from omics sciences and data science, among others

**Table 4:** Proposal of competencies for domain 4: participatory health.

Subdomains		Areas of competence
**Participatory health**		
	D4.1	Information needed to promote the informed participation of patients in shared clinical decision-making (autonomy over their health decisions), taking into account the complexity of the information associated with personalized precision medicine
	D4.2	Contemplate patients’ preferences, taking into consideration the depth with which they want to know the results derived from their health data, diagnostic tests, and treatments
	D4.3	Appropriate communication skills to ensure individualized and quality face-to-face and/or telematic care, secure patient understanding of information, and consider their needs, circumstances (eg, language, culture, socioeconomic status), and expectations
	D4.4	Necessary skills for self-awareness (limits, biases, and external influences) and emotional self-regulation of the professional as a key aspect of humanized care
	D4.5	Needs and demands of patients’ associations to foster their participation as key agents in decision-making at the institutional level

**Table 5:** Proposal of competencies for domain 5: bioethics.

Subdomains			Areas of competence
**Bioethics**			
	D5.1	Principles of bioethics in personalized precision medicine	
	D5.2	Incorporation of ethical aspects in the design of the new health care processes derived from the incorporation of personalized precision medicine into clinical practice	
	D5.3	Functioning and role of the ethics committees and the criteria they use when reaching consensus for the application of personalized precision medicine	
	D5.4	Ethical issues regarding the management and protection of health data, especially in the new scenarios that have arisen in the context of personalized precision medicine	
	D5.5	Patients’ power over their health data, providing the necessary information in a way that, in an informed manner, they can authorize or not its use for biomedical research, contributing to the advancement of personalized precision medicine	

**Table 6:** Proposal of competencies for domain 6: transversal.

Subdomains		Areas of competence
**Management**		
	D6.1	Planning tools, policies, and health regulations linked to the development and implementation of personalized precision medicine in the health system
	D6.2	Health strategies and management tools that would contribute to the implementation of personalized precision medicine at the health care level
	D6.3	Health economics tools to ensure compliance with the principle of equity and promote sustainability in the health system
	D6.4	New developments in the field of personalized precision medicine that imply changes in the organization and/or provision of health care to adapt or develop new health care processes
**Personal development**		
	D6.5	Cross-disciplinary thinking and innovative attitude based on continuous learning to identify improvements and new solutions that contribute to the development of personalized precision medicine
	D6.6	Collaboration and coordination with other professionals as part of a multidisciplinary team recognizing the knowledge and skills of each professional and promoting shared decision-making
	D6.7	Training skills to transfer the knowledge of personalized precision medicine to other health professionals
	D6.8	Critical analysis of information and interpretation of results, understanding the differences between levels of evidence and degrees of recommendation
	D6.9	Health research methods to advance and translate personalized precision medicine to clinical practice, incorporating research as another aspect of their professional work
	D6.10	Communication skills to disseminate scientific advances to citizens and promote their participation in the development of personalized precision medicine
	D6.11	Up-to-date performance of all competencies in the field of personalized precision medicine and the identification of opportunities for improvement in professional practice

### Proposal of Training Levels for Each Competency and Professional Profile

The defined knowledge applies to any health care professional who develops or will develop their professional activity in the field of personalized precision medicine. However, the level of training required for each area of knowledge will depend on their specific profile.

To this end, depending on their professional activity within this field, a classification of progressive levels of development according to the depth that a professional should acquire for each competency was established. Three levels of training were identified: basic, intermediate, and advanced (Textbox 6).

It is important to note that health care professionals will be able to acquire this knowledge at any point during their career through the development and accreditation of training content and programs, as well as certification and recertification systems.

Figures 2-10 display the matrices of the level of competency training by professional profile for each domain.

**Levels of training and description.**

- Basic level: Health care professionals can understand and identify the subject matter and explain the meaning of related information.
- Intermediate level: Health care professionals can apply the knowledge in their daily practice, demonstrating the ability to interpret the information and results and transfer its application to different contexts.
- Advanced level: Health care professionals can integrate knowledge in a complete, consistent, and up-to-date manner, demonstrating the ability to critically analyze and evaluate the results. They are also able to innovate on the knowledge acquired to contribute to the development of personalized precision medicine as part of the medicine of the future.

**Figure 2:**
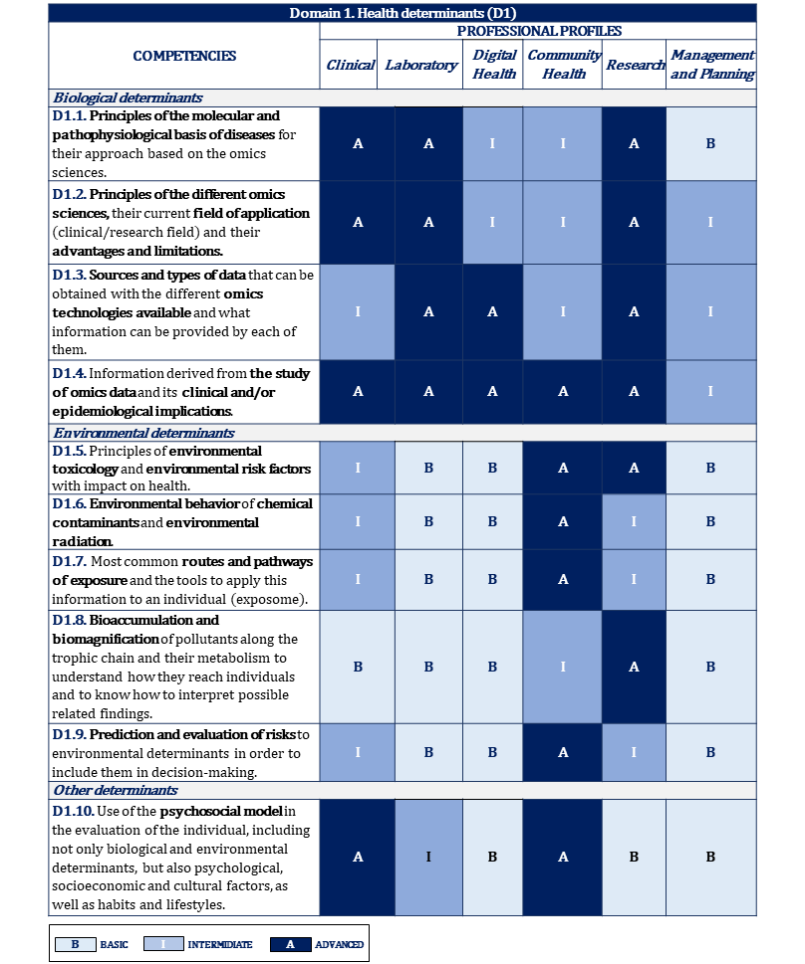
Proposal of competencies training level for domain 1 depending on the professional profile.

**Figure 3:**
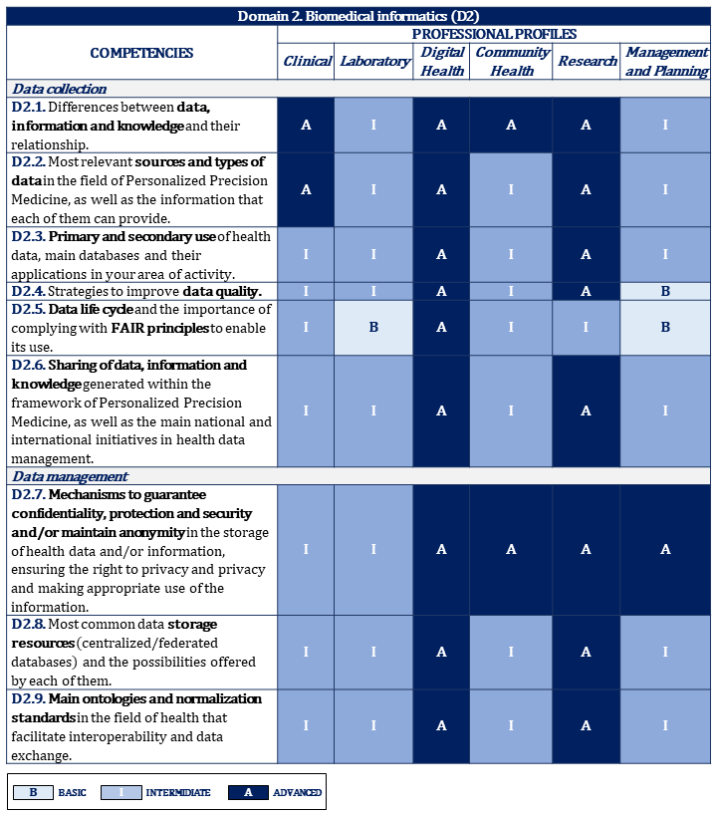
Proposal of competencies training level for domain 2 depending on the professional profile (1/2). FAIR: Findability, Accessibility, Interoperability, and Reusability.

**Figure 4:**
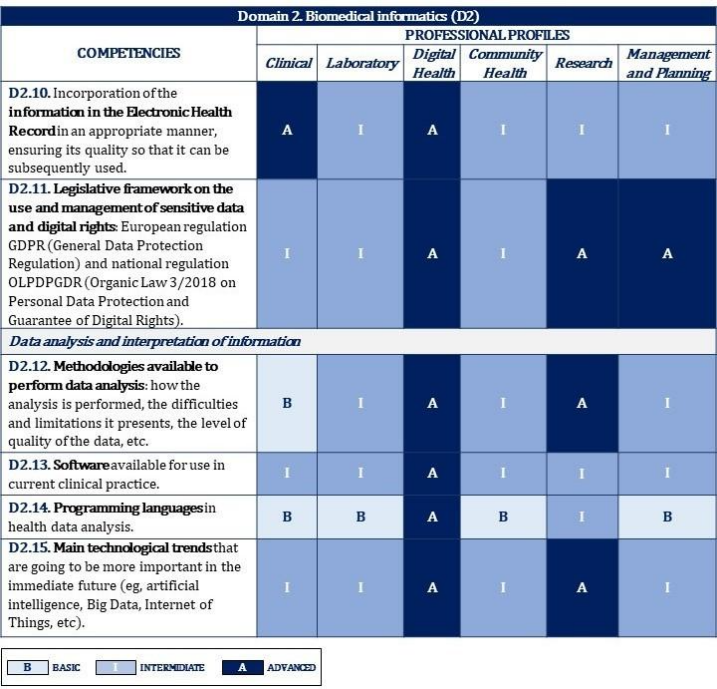
Proposal of competencies training level for domain 2 depending on the professional profile (2/2).

**Figure 5:**
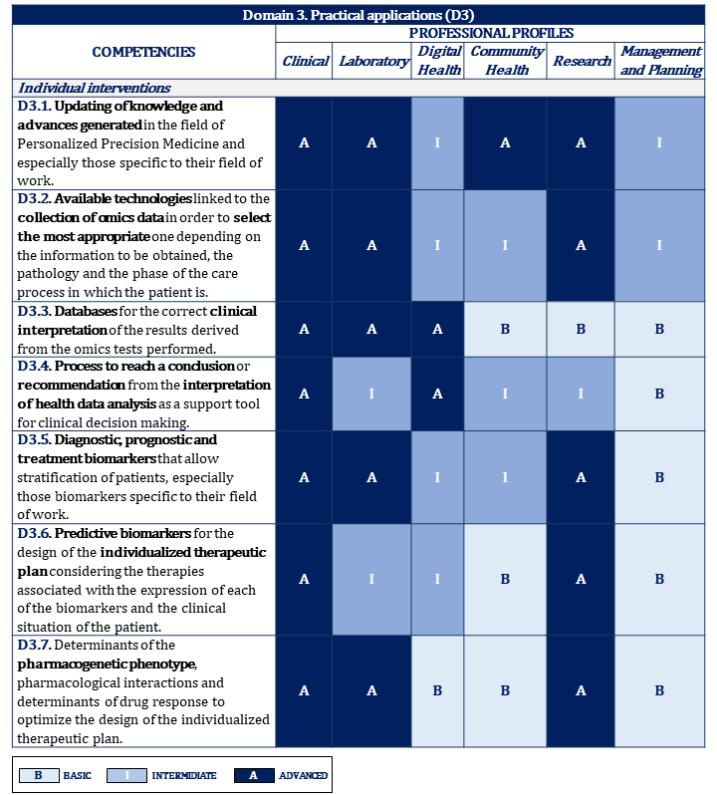
Proposal of competencies training level for domain 3 depending on the professional profile (1/2).

**Figure 6:**
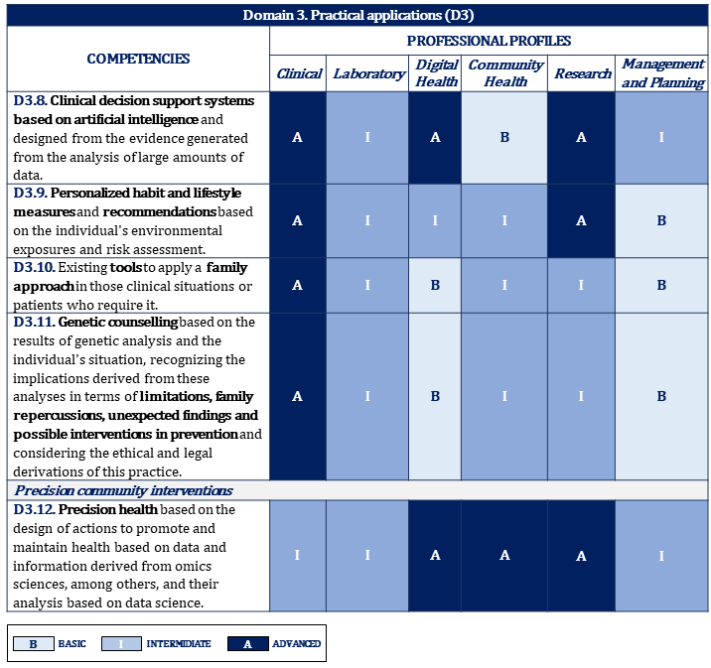
Proposal of competencies training level for domain 3 depending on the professional profile (2/2).

**Figure 7:**
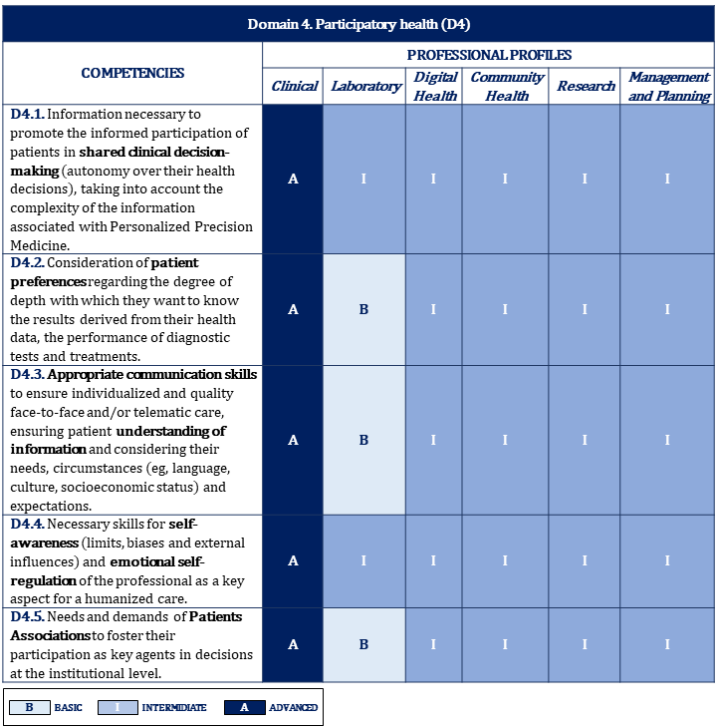
Proposal of competencies training level for domain 4 depending on the professional profile.

**Figure 8:**
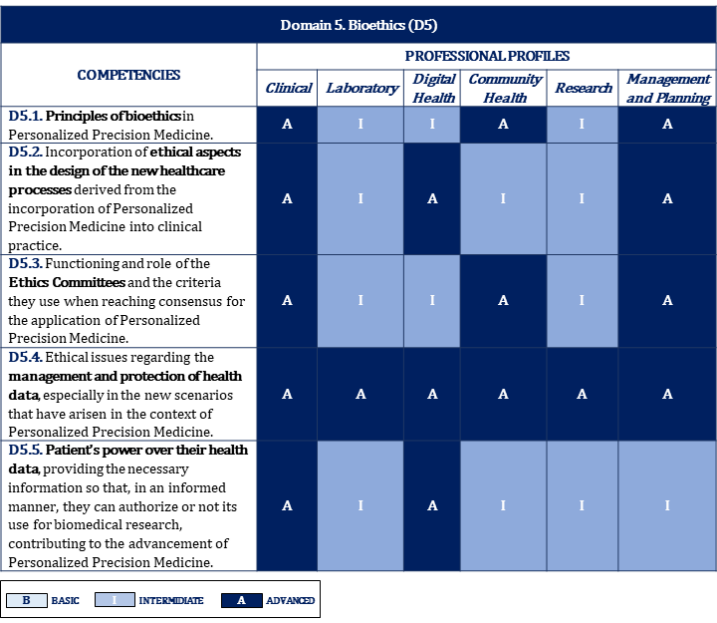
Proposal of competencies training level for domain 5 depending on the professional profile.

**Figure 9:**
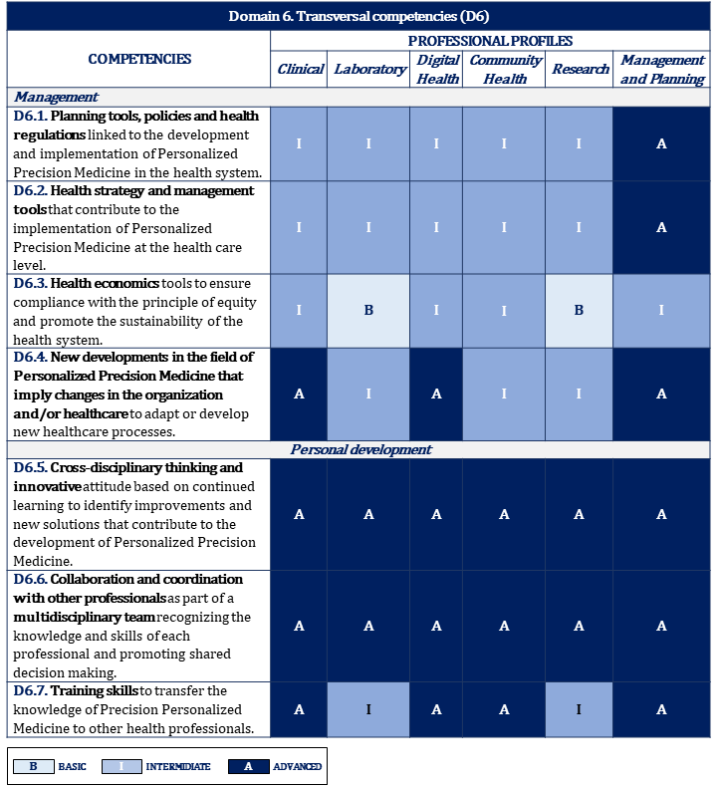
Proposal of competencies training level for domain 6 depending on the professional profile (1/2).

**Figure 10:**
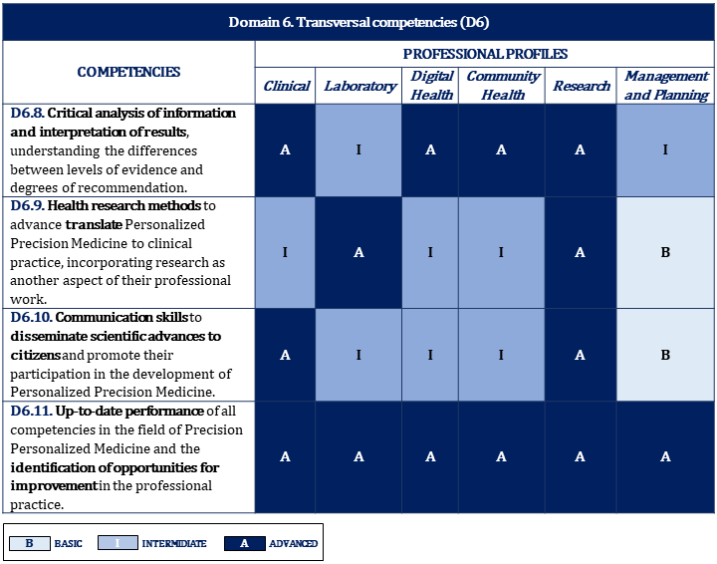
Proposal of competencies training level for domain 6 depending on the professional profile (2/2).

## Discussion

The elaboration of this framework has been carried out by taking into account other competence frameworks previously defined by national and international scientific organizations [4,5,9-25,27,28,70,71]. Therefore, a common structure has been followed, establishing basic and transversal competencies within each of the domains. After the analysis of documents and with the opinion of the experts, 58 competencies were defined and structured into 5 essential domains: health determinants, biomedical informatics, practical applications, participatory health, and bioethics, along with a cross-cutting domain that impacts the overall performance of the competencies linked to each of the domains. It should be noted that the most relevant areas of knowledge that will shape the future of health care, such as omic sciences or artificial intelligence, are included within the framework. Thus, this framework defines a proposal of essential domains and common competencies for all health care professionals necessary to optimally develop their professional practice in personalized precision medicine as a fundamental part of the medicine of the future.

Likewise, 6 generic professional profiles were identified and defined according to the area where they carry out their professional activity: clinical, laboratory, digital health, community health, research, and management and planning. To adapt to new professionals that may arise from the integration of personalized precision medicine into the health care system, those that emerge from the digital transformation of the health care system have been included, as in the case of the digital health profile. Additionally, although all professionals must have a common background, having at least a basic knowledge of all domains and competencies, each competency was classified by progressive levels of training (basic, intermediate, and advanced) according to the required skills and functions of the professional profile.

Considering the progress and integration of personalized precision medicine within the health care system, this proposal of competencies represents a turning point in the training of professionals who carry out their work in this emerging field of medicine, providing high-quality, personalized health care that considers the individual circumstances and implications of all patients. This competency framework will serve as an instrument to support the development and implementation of training and certification programs for health care professionals working in personalized precision medicine. Finally, to guarantee its usefulness over time, the competency framework has been designed as a dynamic document that can adapt to the changes that will occur with the advancement of this field.
