# Correction: Personalized Precision Medicine for Health Care Professionals: Development of a Competency Framework

**DOI:** 10.2196/46366

**Published:** 2023-02-21

**Authors:** Fernando Martin-Sanchez, Martín Lázaro, Carlos López-Otín, Antoni L Andreu, Juan Cruz Cigudosa, Milagros Garcia-Barbero

**Affiliations:** 1 Department of Biomedical Informatics and Digital Health National Institute of Health Carlos III Madrid Spain; 2 Department of Medical Oncology, University Hospital Complex of Vigo Vigo Spain; 3 Department of Biochemistry, University of Oviedo Oviedo Spain; 4 European Infrastructure for Translational Medicine Amsterdam Netherlands; 5 Department of University, Innovation and Digital Transformation, the Government of Navarra Navarra Spain; 6 Faculty of Medicine, Miguel Hernández University Alicante Spain

In “Personalized Precision Medicine for Health Care Professionals: Development of a Competency Framework” (JMIR Med Educ 2023;9:e43656), the authors noted several errors.

In the originally published paper, a typographical error in the corresponding author’s email address, formatted as:

fmartin@isciiI.es

This has been changed to:

fmartin@isciii.es

In the last paragraph of the “Introduction” section, the authors noted that one reference was not included in the original manuscript. A new citation (numbered 7) has been added to the list of references and cited in the following sentence:

Accordingly, this project aimed to define a proposal of common domains and competencies for today’s health care professionals, as well as those who will emerge in the future [7].

The citation added to the reference list is as follows:

Fundación Instituto Roche. Competency Personalized Precision Medicine for healthcare professionals. 2021. URL: https://www.institutoroche.es/static/pdfs/ Final_Report_Competencies_PPM_DEF1.pdf [accessed 2023-02-08]

The addition of this citation modified the order of the rest of the citations.

In Table 2, an acronym was originally typed incorrectly as “OLPPDGDR”. This has been corrected to “OLPDPGDR” both in row “D2.11” and in the corresponding footnote “c”. The same error occurred in Figure 4, which has also been updated accordingly.

An error was also noted in the first paragraph of the “Acknowledgments” section. The sentence:

We are grateful to the working group for aiding the development of this project, contributing to the preparation of this document, and sharing their perspectives on the key elements and training needs for the definition of competencies in the areas of interest of personalized precision medicine.

Has been changed to:

We are grateful to the Fundación Instituto Roche and the working group for aiding the development of this project, and sharing their perspectives on the key elements and training needs for the definition of competencies in the areas of interest of personalized precision medicine.

The correction will appear in the online version of the paper on the JMIR Publications website on February 21, 2023, together with the publication of this correction notice. Because this was made after submission to full-text repositories, the corrected article has also been resubmitted to those repositories.
